# Living to Purpose

**Published:** 1861-10

**Authors:** 


### HALL'S JOURNAL OF HEALTH.

WE AIM TO SHOW HOW DISEASE MAY BE AVOIDED, AND THAT IT IS BEST, WHEN SICKNESS COMES, TO TAKE NO MEDICINE WITHOUT CONSULTING A PHYSICIAN.

Vol. VIII.] OCTOBER, 1861. [No. 10.

### LIVING TO PURPOSE.

NEARLY a hundred years ago, there lived a young man on the frontiers of Virginia, without money, and without a name, dependent on his daily labor for a living; and in the absence of any other special aim in life, he concluded to undertake to educate, at his own expense, a youth who seemed to him to be one of more than ordinary promise. What were the thoughts of Gideon Richie, when plowing, and hoeing corn, and chopping wood, and mauling rails; what visions of the future he indulged in during the hours of weary labor, we may never know. He must have covered a warm heart, and a high purpose, and a stern resolve, in that homespun dress of wool, and moccasin, and hunting-shirt, which characterized those who lived on the farthest frontiers of a semi-civilization; for he worked on, without faltering, until he saw his *protégé* a minister of the gospel, who rose like a star in the western firmament, casting its beams of light into the wigwam of the Indians of the West, and away back again into the saloons of the *élite* about "Boston Common." Young Richie died, and but for the shining of his adopted son, his name would long since have passed from the memory of man. But he was placed here for a purpose, in the providence of God; and having answered that purpose with a will, his heart being in the right place, he has, doubtless, gone up higher, for an enduring reward among the blessed. Had he been an unwilling instrument, still the purpose would have been subserved in some way, but he would have lost the reward.

The young minister became the founder of churches, and schools, and academies. Now, a leader of the soldiers of his country, and then of soldiers of the cross; now, at the head of a church, then at the head of a college. Now, as we have heard him say, banqueting with the merchant princes of the East; then, wrapped in his saddle blanket, sleeping across logs of wood, while deluging rains were driving their gathering currents under him in the wilderness of the savage. Now, the benignant listener to the religious experiences of the Indian and the Negro; then, himself the listened to, by wrapt thousands, as they looked to the gestures of his pointed finger, or hung upon entrancing words as they fell from his lips. His heart so stern, that, like his eagle eye, it never quailed before mortal man; and yet of such womanly softness, that there was a well-spring of tears within it, which overflowed at the first cry of dependence or of pity. In a contest, face to face, with the old hero of the Hermitage, of might with right, even General Jackson was the vanquished, and Gideon Blackburn became the acknowledged conqueror. Of the hundreds, if not thousands, of young men whom Dr. Blackburn has aided by his teachings, his counsels, and his money, to reach the ministry, not a man of them now living is there, who will not rise up and call his memory blessed. Of his pupils at college, who have been, or are to-day, in the high places of law, medicine, and divinity—as Governors of States, or Members of Congress; as professors or presidents in academies, colleges, and universities—there is not a man of them who can, by any possibility, look backward thirty years, and not remember in Dr. Blackburn the personification of the patriarch, the man, the Christian gentleman. The last work of his life was the establishment of a Theological Seminary in the West, known by his name, and which bids fair to be a fountain from which streams of ministers shall flow, to found, and feed, and fructify churches, until the end of time. Man of immortality, mortal of an hour, yet destined, by your acts, to exert influences on the world for all time—influences for good or for evil—for happifying your race, or for degrading it—if you can, by any work, save a dime or two a day, go this moment, and resolve to be another Gideon Richie, and raise another Blackburn!

Young man, fatherless, motherless, penniless, wake up, and remember, you may be a Blackburn, too!

